# Survival and swimming behavior of insecticide-exposed larvae and pupae of the yellow fever mosquito *Aedes aegypti*

**DOI:** 10.1186/1756-3305-7-195

**Published:** 2014-04-24

**Authors:** Hudson VV Tomé, Tales V Pascini, Rômulo AC Dângelo, Raul NC Guedes, Gustavo F Martins

**Affiliations:** 1 Departamento de Entomologia, Universidade Federal de Viçosa, Viçosa, MG 36570-900, Brazil; 2 Departamento de Biologia Geral, Universidade Federal de Viçosa, Viçosa, MG 36570-900, Brazil

**Keywords:** *Aedes aegypti*, Behavioral response, Biopesticides, Dengue vector, Mosquito, Sublethal toxicity

## Abstract

**Background:**

The yellow fever mosquito *Aedes aegypti* is essentially a container-inhabiting species that is closely associated with urban areas. This species is a vector of human pathogens, including dengue and yellow fever viruses, and its control is of paramount importance for disease prevention. Insecticide use against mosquito juvenile stages (i.e. larvae and pupae) is growing in importance, particularly due to the ever-growing problems of resistance to adult-targeted insecticides and human safety concerns regarding such use in human dwellings. However, insecticide effects on insects in general and mosquitoes in particular primarily focus on their lethal effects. Thus, sublethal effects of such compounds in mosquito juveniles may have important effects on their environmental prevalence. In this study, we assessed the survival and swimming behavior of *A. aegypti* 4^th^ instar larvae (L4) and pupae exposed to increasing concentrations of insecticides. We also assessed cell death in the neuromuscular system of juveniles.

**Methods:**

Third instar larvae of *A. aegypti* were exposed to different concentrations of azadirachtin, deltamethrin, imidacloprid and spinosad. Insect survival was assessed for 10 days. The distance swam, the resting time and the time spent in slow swimming were assessed in 4^th^ instar larvae (L4) and pupae. Muscular and nervous cells of L4 and pupae exposed to insecticides were marked with the TUNEL reaction. The results from the survival bioassays were subjected to survival analysis while the swimming behavioral data were subjected to analyses of covariance, complemented with a regression analysis.

**Results:**

All insecticides exhibited concentration-dependent effects on survival of larvae and pupae of the yellow fever mosquito. The pyrethroid deltamethrin was the most toxic insecticide followed by spinosad, imidacloprid, and azadirachtin, which exhibited low potency against the juveniles. All insecticides except azadirachtin reduced L4 swimming speed and wriggling movements. A similar trend was also observed for swimming pupa, except for imidacloprid, which increased the swimming activity of pupa. Curiously, the insecticides did not affect cell damage in the neuromuscular system of larvae and pupae.

**Conclusions:**

Deltamethrin and spinosad were the main compounds to exhibit lethal effects, which allowed the control of *A. aegypti* larvae and pupae, and impair their swimming potentially compromising foraging and predation likelihood.

## Background

The yellow fever mosquito *Aedes aegypti* is a container-inhabiting species that is closely associated with urban areas. This species is a vector of human pathogens, including dengue and yellow fever viruses. Suitable human environments are prevalent in tropical countries, where dengue is recognized as one of the most devastating vector-borne diseases [1]. The main control strategies currently used against dengue virus transmission still focus on managing its vector populations, and insecticide use is prevalent in this scenario [2-4].

Neurotoxic insecticides, particularly organophosphate and pyrethroids, are the most frequently used compounds against adults of *A. aegypti*. However, these compounds feature increasing problems of insecticide resistance in Asia and Latin America and increased concerns for human safety [5-7]. Consequently, insecticide use against mosquito juveniles is growing in importance, but this use is potentially afflicted by the same shortcomings of insecticides that target adults and thus requires alternative compounds. In addition to the neonicotinoid imidacloprid, several other compounds have been tested against mosquitoes, including biopesticides, such as azadirachtin, a terpenoid mixture obtained from the neem tree (*Azadirachta indica* A. Juss.), and spinosad, a mixture of secondary metabolites obtained as fermentation products from the soil actinomycete *Saccharopolyspora spinosa*[8-13].

Insecticide effects on insects in general and mosquitoes in particular focus mainly on their lethal effects [2,14]. However, the sublethal effects of such compounds applied against mosquito larva and pupa may have important effects on crucial insect activities, and insecticide degradation will invariably lead to the sublethal exposure of a target (and non-target) species, which requires the assessment of such effects [15-18]. Several activities performed by mosquito juveniles, such as breathing, foraging, refuge seeking and predator evasion, are strictly dependent on swimming, which emphasizes the importance of insecticide-induced changes in such (swimming) behavioral patterns to the dynamics of the mosquito population [19-23].

In this study, we assessed the survival and swimming behavior of 4^th^ instar larvae (L4) and pupae of the yellow fever mosquito *A. aegypti* exposed to increasing concentrations of the insecticides azadirachtin, deltamethrin, imidacloprid and spinosad. High lethal efficacy was expected for these compounds, of which deltamethrin is frequently used against the adults and larvae of *A. aegypti*, based on results reported for different mosquito species [12,13,24-28]. Although not yet explored, we also expected increased cell damage in the neuromuscular system of insecticide-exposed L4 and swimming impairment in L4 and pupae exposed to sublethal concentrations of the tested compounds.

## Methods

### Insects and insecticides

*Aedes aegypti* (strain PP-Campos, Campos dos Goytacazes, RJ, Brazil) were obtained from a colony maintained in the Department of General Biology of the Federal University of Viçosa (Viçosa, MG, Brazil). The larvae were maintained in dechlorinated tap water and fed turtle food daily (Reptolife, Alcon Pet, Camburiú, SC, Brazil) under controlled temperatures (25 ± 2°C), relative humidities (60 ± 2%), and photoperiods (12:12 L:D).

The four insecticides (and respective commercial formulations) used in the experiments were azadirachtin (Azamax, 12 g a.i./L, emulsifiable concentrate, DVA Brasil, Campinas, SP, Brazil), deltamethrin (Decis 25CE, 25 g a.i./L, emulsifiable concentrate, Bayer CropScience, São Paulo, SP, Brazil), imidacloprid (Evidence WG, 700 g a.i./L, water dispersible granule, Bayer CropScience, São Paulo, SP, Brazil), and spinosad (Tracer EC; 480 g a.i./L, concentrated suspension, Dow AgroScience, Santo Amaro, SP, Brazil). Deltamethrin is representative of the pyrethroid insecticides of common use against mosquito larvae and pupae, while azadirachtin, imidacloprid and spinosad are potential alternative compounds for pyrethroids and older organophosphates used against mosquitos.

The insecticides were diluted in distilled and deionized water to obtain the desired concentrations used in the experiments. Deltametrin was poured into the plastic containers after initial dilution to prevent reaction of the eventual organic solvents from the formulation with the plastic of the container.

### Survival bioassays

Batches (replicates) of 25 insects (3^rd^ instar larvae; L3) were placed in 500 mL plastic containers filled with 200 mL clear water (distilled and deionized; with or without insecticide) and 10 mg of turtle food. Four batches (replicates) were used for each concentration and each insecticide in addition to a control without insecticide (i.e. containing only clear water).

Insect survival was assessed daily for 10 days, which is sufficient for the insects to reach the adult stage. The insects were considered dead if they were unable to move when prodded with a fine hairbrush, after which they were removed from the test containers. The number of dead insects divided by the initial number of insects provided the survivorship values necessary for the survival analysis; the dead insects were not replaced. The insecticide concentrations used were 0.0, 0.1, 0.5, 1.0 and 10.00 ppm azadirachtin; 0.0, 0.001, 0.01, 0.05, 0.1, 0.5, 1.0 and 10.0 ppm deltamethrin; 0.0, 0.15, 1.5, 3.0, 6.0 and 15.0 ppm for imidacloprid; and 0.0, 0.025, 0.05, 0.1, 0.5, 1.0, 4.0 and 10.0 ppm for spinosad. Different concentrations of each insecticide were used to demonstrate the concentration-dependent effects on survival and all behavioral parameters were assessed.

### Swimming bioassay

Fourth instar larvae (L4; 24 h after exposure of 3^rd^ instar larvae to insecticides) and one-day old pupae (96 h after exposure of the 3^rd^ instar larvae) were used to assess the swimming behavior of the insecticide-exposed insects. The developmental stages allow more consistent and detailed determinations besides those also commonly targeted by insecticide applications. Each insect was individually transferred from the insecticide-contaminated containers to a Petri dish arena (9 cm diameter and 2 cm high) filled with clear water (at a height of 1 cm) free of insecticides. The swimming activity of each insect was recorded for 15 min with a charge-coupled device camera (CCD) and digitally transferred to a computer equipped with video-tracking software (VideoTrack System, Viewpoint LifeSciences, Montreal, Canada). The camera was positioned 30 cm from the arena, and the water in the arena was replaced after each recording. The parameters assessed were distance swam (cm), the resting time (s) and the time spent in slow swimming (s). A threshold of 0.6 cm/s was used to distinguish slow from fast swimming, and the swimming tracks below this threshold are depicted in green, while those above this threshold are depicted in red. Twenty larvae and 20 pupae were used for each concentration of each insecticide, which resulted in significant insect survival at these developmental stages. The swimming bioassays were carried out between 2-6:00 pm under incandescent light and at a temperature of 25 ± 2°C.

### Cell death in the neuromuscular system

Five larvae (L4; 24 h after exposure) and five pupae (one-day old; 96 h after exposure) of the same previous insecticidal treatments were collected and subjected to an assessment of cell death of muscular and nervous cells associated with the central nervous system, which is associated with swimming. The abdomens were dissected in insect physiological solution (0.1 M phosphate buffer under pH 7.4) and subsequently fixed for 24 h in 2.5% glutaraldehyde in 0.1 M cacodylate buffer (pH 7.4).

The fixed material was dehydrated in a crescent ethanol solution (70-100%) and embedded overnight in historesin Leica® (Heidelberg, Germany). The sample was subsequently embedded in historesin with hardener and subjected to microtomy. Five μm slices were transferred to glass slides and treated with proteinase K [10 μM/mL of Tris-HCl (10 mM, pH 7.4)] for 1 h at 37°C. The slides were washed three times in 0.1 M phosphate buffer (pH 7.4) and subsequently marked with the TUNEL reaction (Roche Aplied Science, Penzberg, Germany) for 1 h at 37°C. The slides were subsequently washed and covered with anti-fading media (Mowiol, Sigma-Aldrich Brasil, São Paulo, SP, Brazil). The slides exhibiting the cells under study were analyzed under a fluorescence microscope using a WU filter (BX60, Olympus, Center Valley, PA, USA).

### Statistical analyses

The results from the survival bioassays were subjected to survival analysis using the procedure LIFETEST from SAS (SAS Institute, 2008), in which survival curves are obtained using Kaplan-Meyer estimators for insects exposed to each insecticide concentration. The insects reaching the adult stage were treated as censored data. The swimming behavioral data were subjected to analyses of covariance (ANCOVA) with the behavioral traits as dependent variables, the insecticide as the independent variable and the insecticide concentration as the covariate (procedure GLM; SAS Institute [29]). The analysis of covariance was complemented with regression analyses when appropriate (procedure REG; SAS Institute [29]). All data were checked for the homogeneity of variance and normality (PROC UNIVARIATE, PROC GPLOT [29]), and data transformation was not necessary.

## Results

### Survival analyses

**Figure 1:**
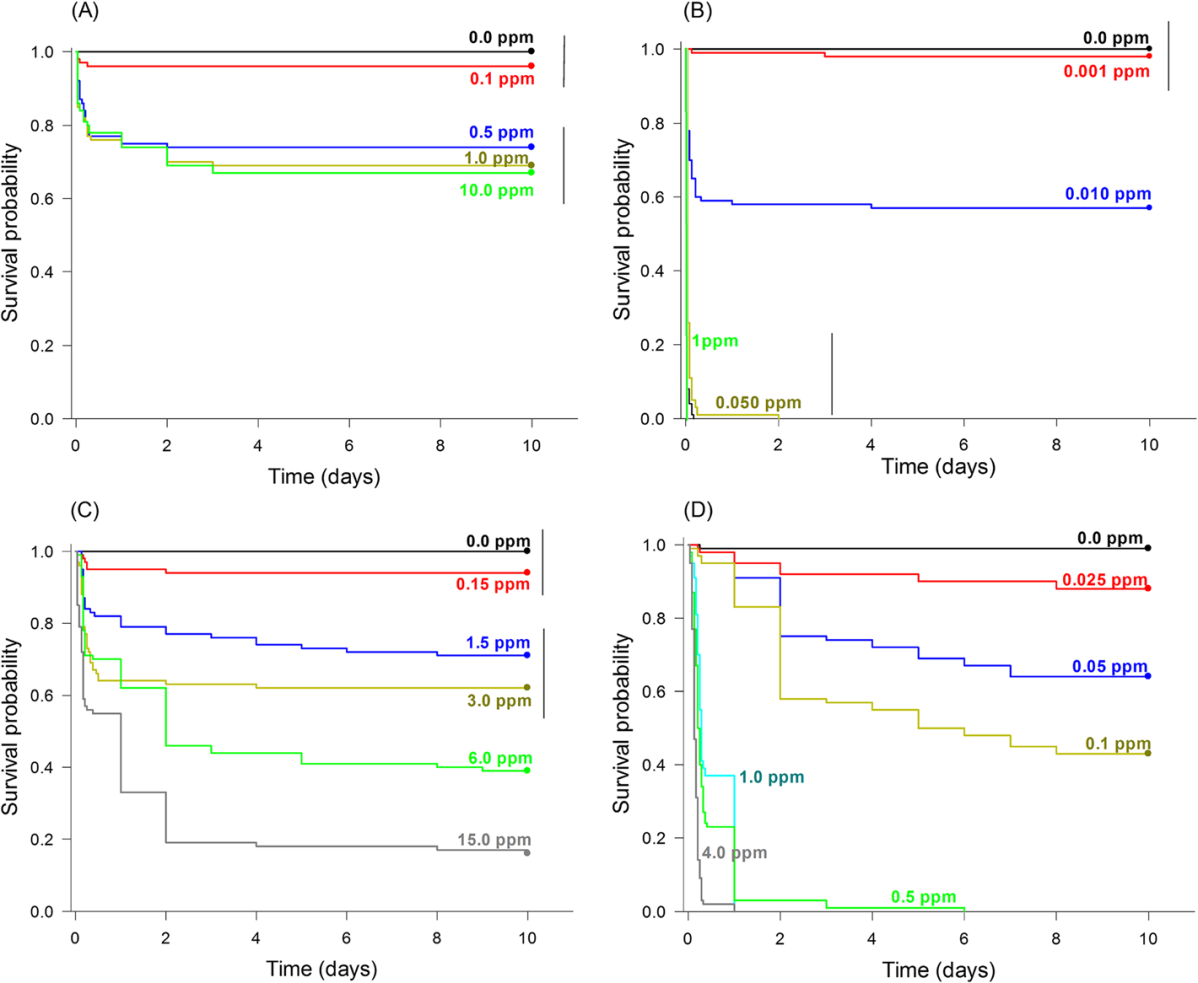
**Survival curves of larvae of *A. aegypti *exposed to azadirachtin (A), deltamethrin (B), imidacloprid (C), and spinosad (D).** Survival curves grouped by the same line are not significantly different by Holm-Sidak’s test (*p* > 0.05).

Survival (time-mortality response) differed significantly among the concentrations of each insecticide, namely azadirachtin (Log-rank test, χ^2^ = 58.20, df = 4, *p* < 0.001), deltamethrin (Log-rank test, χ^2^ = 382.66, df = 7, *p* < 0.001), imidacloprid (Log-rank test, χ^2^ = 238.65, df = 5, *p* < 0.001), and spinosad (Log-rank test, χ^2^ = 837.94, df = 7, *p* < 0.001) (Figure 1). Deltamethrin was particularly potent against *A. aegypti* L4, followed by spinosad, imidacloprid and azadirachtin, which provided only 30% efficacy at its highest concentration (10.0 ppm).

### Swimming activity

**Figure 2:**
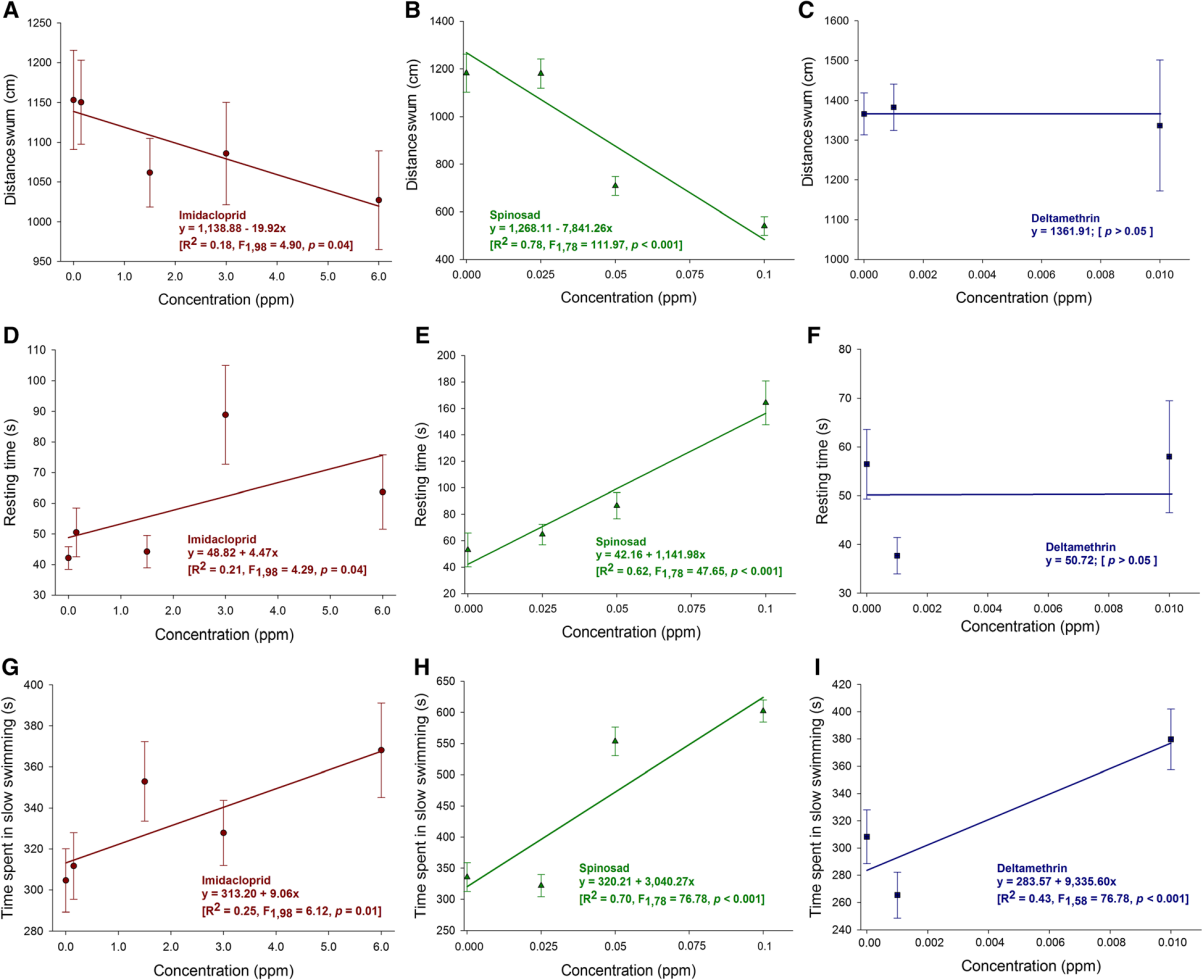
**Linear regressions showing variation of distance swum, resting time and time spent in slow swimming in clear water (< 0.6 cm/s) of L4 of *A. aegypti *after 24 h of insecticide exposure.** Panels **A**, **D** and **G**: L4 exposed to imidacloprid. Panels **B**, **E** and **H**: L4 exposed to spinosad. Panels **C**, **F** and **I**: L4 exposed to deltametrin.

**Figure 3:**
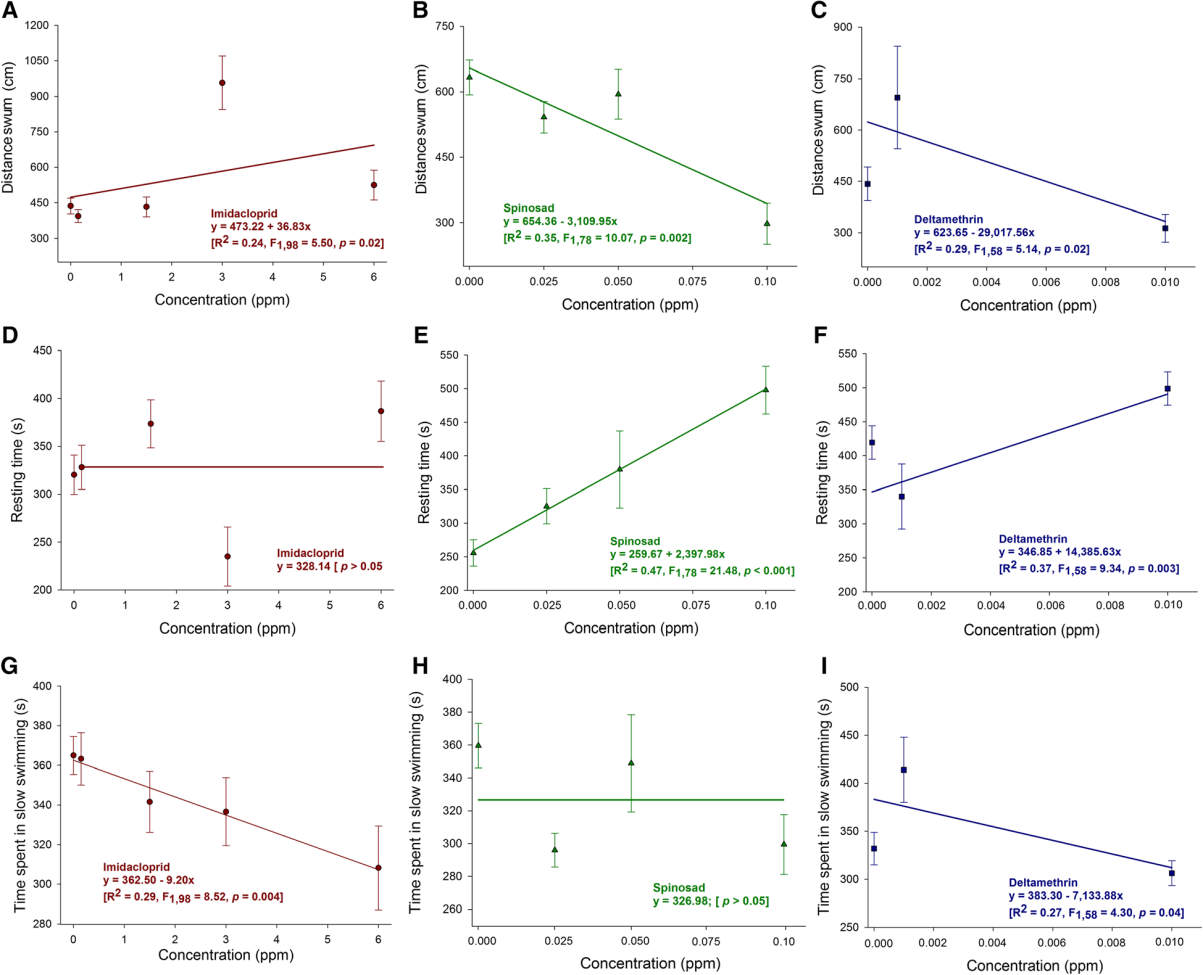
**Linear regressions showing variation of distance swum, resting time and time spent in slow swimming in clear water (< 0.6 cm/s) of pupae of *A. aegypti *after 96 h of insecticide exposure.** Panels **A**, **D** and **G**: pupae exposed to imidacloprid; Panels **B**, **E** and **H**: pupae exposed to spinosad; Panels **C**, **F** and **I**: pupae exposed to deltametrin.

The swimming activity of L4 and pupae of *A. aegypti* exhibited significant concentration-dependent differences among insecticides. The interaction insecticide-concentration was significant in the analyses of covariance carried out for each of the parameters assessed for both L4 and pupae (Table 1). The complementary regression analyses indicated a concentration-dependent reduction in the distance swum (Figure 2A), an increase in the resting time (Figure 2D) and slow swimming (Figure 2G) of the L4 for imidacloprid. Similarly regression analyses indicated a concentration-dependent reduction in the distance swum (Figure 2B), an increase in the resting time (Figure 3E) and slow swimming (Figure 2H) of the L4 for spinosad, and an increase in slow swimming for deltamethrin (Figure 2I). However, increasing concentrations of deltamethrin did not affect the distance swum (Figure 2C) nor the resting time (Figure 2F). Increasing concentrations of azadirachtin did not affect any of the assessed swimming traits (distance swum: 1.227.61 ± 103.09 cm; resting time: 53.38 ± 13.04 s; time in slow swimming: 340.20 ± 25.74 s) (*p* > 0.05).

**Table 1:** **Results of the analyses of covariance for the swimming behavior of larvae and pupae of the yellow fever mosquito *A. aegypti *** **after 24 h and 96 h of insecticide exposure**

**Developmental stage**	**Sources of variation**	**Swimming distance (cm)**		**Resting time (s)**		**Time spent in slow swimming (s)**	
		**F**	***p***	**F**	***p***	**F**	***p***
4^th^ instar larvae	Model	7.62	< 0.001	5.32	< 0.001	18.01	< 0.001
	Insecticide (I)	6.93	< 0.001	6.82	< 0.001	11.97	< 0.001
	Concentration (C)	4.55	< 0.001	3.29	< 0.001	15.96	< 0.001
	Interaction I x C	5.04	0.025	21.03	< 0.001	4.49	0.035
Pupae	Model	4.90	< 0.001	5.43	< 0.001	3.57	< 0.001
	Insecticide (I)	0.66	0.58	2.33	0.074	0.67	0.569
	Concentration (C)	5.85	< 0.001	4.34	< 0.001	3.75	< 0.001
	Interaction I x C	2.60	0.11	11.78	< 0.001	6.19	0.013

Imidacloprid exposure increased the pupae swimming distance (Figure 3A). In contrast, a decreasing trend was also apparent for the pupae swimming distance in response to spinosad (Figure 3B) and deltamethrin (Figure 3C), with azadirachtin also not exhibiting a significant concentration-dependent response (*p* > 0.05; 447.22 ± 68.43 cm). Pupal resting time increased in response to spinosad (Figure 3E) and deltamethrin (Figure 3F) but was not affected by imidacloprid (Figure 3D) and azadirachtin (not shown) irrespective of their concentration (*p* > 0.05; 415.55 ± 35.35 s). Slow swimming activity decreased in response to increased concentrations of imidacloprid (Figure 3G) and deltamethrin (Figure 3I), while spinosad and azadirachtin did not affect this trait (*p* > 0.05; Figure 3H and 340.20 ± 25.74, respectively).

**Figure 4:**
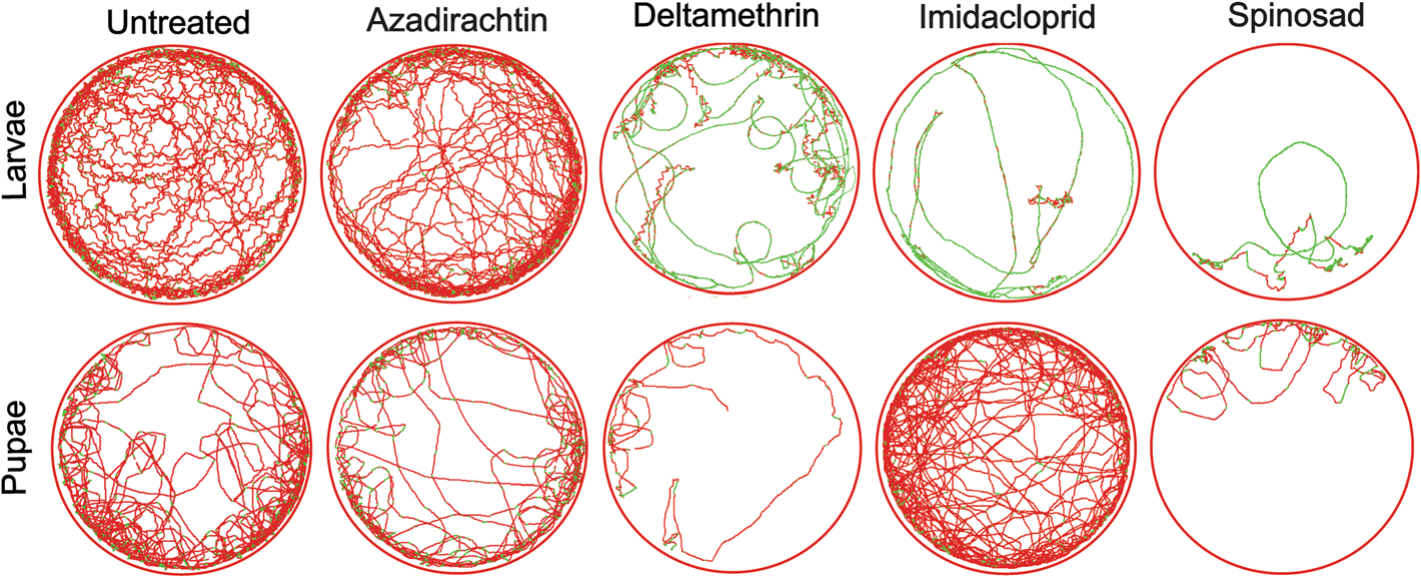
**Representative swimming tracks of larvae and pupae of the yellow fever mosquito in clear water after 24 h and 96 h of insecticide exposure, respectively.** Red tracks represent fast swimming (> 0.6 cm/s), while green tracks represent slow swimming of individual insects (< 0.6 cm/s).

The larvae of the yellow fever mosquito swim by means of wriggling movements, with insects flexing their bodies from one side to the other to exhibit a zig-zag tracking pattern that is characteristically detected in the untreated L4 (Figure 4). However, the L4 did not exhibit this wriggling swimming pattern when exposed to deltamethrin, imidacloprid and spinosad. In response to exposure to these compounds, the L4 swam mainly in a non-wriggling straight pattern, which likely reduced the swimming velocity (represented in green in Figure 4). The prevalence of the green tracks (slow movement; < 0.6 cm/s) over red tracks (fast movement; > 0.6 cm/s) is associated with the concentration of these insecticides. The distance swum reduced with insecticide exposure, which reduced the number of swimming tracks (Figure 4).

### Cell death in the neuromuscular system

Despite the detected changes in the swimming behavior of L4 and pupae of mosquitos in response to insecticide exposure, no significant death of muscle cells associated with the motor system was observed. The same result was obtained for neurons of the central nervous system of mosquitoes.

## Discussion

Because insecticide resistance problems have escalated in populations of *A. aegypti* subjected to the use of adult-targeted compounds, insecticide use against the juvenile stages of this mosquito is becoming increasingly important. The pyrethroid deltamethrin is one of the main compounds used in water against mosquito larvae and pupae, but it also features the problem of increasing insecticide resistance [6,7]. Therefore, the search for alternative insecticides against mosquito juveniles has been receiving increasing attention, and biopesticides, such as azadirachtin and spinosad, have been highly regarded and deemed potentially useful against mosquitos [9,11-13]. The high lethal efficacy of deltamethrin and spinosad against the larvae of the yellow fever mosquito observed in our study largely confirmed this expectation. Azadirachtin and imidacloprid did not exhibit this efficacy.

Concentrations as low as 0.5 ppm of deltamethrin and spinosad led to 100% larvae mortality in less than five days. In contrast, concentrations as high as 15 ppm imidacloprid still allowed nearly 20% survival after 10 days of exposure, and 10 ppm azadirachtin allowed a little less than 70% survival during this timeframe, which indicated a rather poor efficacy against mosquito larvae. These results greatly differ from those reported by Dua *et al.*[12] and Maheswaran and Ignacimuthu [13], which may be due to differences in the testing methodology or, more likely, differences in the formulations used. The standardization, adjuvants and concentration of the active ingredient are frequent problems in the use of botanical biopesticides (i.e. obtained from plant extracts), such as azadirachtin [30,31].

Antonio-Arreola *et al.*[27] also reported a high efficacy of imidacloprid; specifically, they reported a 99% mortality of mosquito larvae exposed to 0.15 ppm of this neonicotinoid insecticide for 24 h, while we did not observe more than 5% mortality at this concentration. Differences in the susceptibility may exist between these populations, which should be considered. Moreover, differences in the methodology (i.e. insect developmental stage, environmental conditions etc.) may have contributed to the distinct results. For instance, the food provision in our bioassays may have provided energy resources to allow the larvae to express their full detoxification potential and increase their tolerance to the insecticides in general and imidacloprid in particular [23].

In addition to its low lethal efficacy, azadirachtin did not impair the swimming of mosquito juveniles, unlike the neurotoxic insecticides tested. This finding was a surprise because azadirachtin interferes with neuroendocrine regulation, which frequently leads to incomplete molt, longer developmental time and malformation [32,33]. However, the low-potency lethal efficacy of azadirachtin is consistent with the lack of sublethal effect observed in the swimming behavior of juveniles and the lack of morphological changes in the exposed insects from our study.

Deltamethrin, imidacloprid and spinosad all compromised mosquito juvenile swimming in response to sublethal exposure. Deltamethrin and spinosad were particularly potent impairing juvenile swimming as indicated by the steep slopes and low concentrations of these insecticides compromising the swimming activity of mosquito larvae and pupae. Impairment of swimming is expected for neurotoxic insecticides [8,26,34,35]. Several studies recorded increased swimming activity, which led to a higher risk of predation in response to insecticide exposure [22,23]. In these studies, the swimming activity was assessed after a short exposure time to detect an initial hyper-excitability associated with sodium-channel modulators (such as pyrethroids) and agonists and modulators of nicotinic acetylcholine receptors (imidacloprid and spinosad, respectively) [26,36-38]. In our study, swimming behavior was recorded after a longer time of exposure, and the inhibitory effects caused by the neurotoxic insecticides tested prevailed, with the sole exception of imidacloprid-exposed pupae (where insecticide exposure favored higher swimming activity).

The wriggling swimming characteristic of mosquito larvae drastically changed in response to neurotoxic insecticide exposure [39,40]. Non-wriggling swimming, i.e. a more straight moving pattern and slow locomotion, prevailed after exposure to deltamethrin, imidacloprid and spinosad. The mouth brushes of the larval mouth parts, apparently associated with feeding, are the main drivers of this straight, forward larval swimming [19], and the prevalence of this swimming movement may compromise feeding, refuge seeking and escape responses. Wriggling swimming does not occur in mosquito pupae [40], but their exposure to the neurotoxic insecticides reduced swimming activity while increasing the resting time, particularly for deltamethrin and spinosad. The differences in the swimming response to imidacloprid in L4 and pupa (reducing the former and increasing the latter) may be due to the prevailing subtype of nicotinic acethylcoline receptor in each of these developmental stages [36], and other factors that lead to differential rate of insecticide penetration and/or detoxification in the insect developmental stages, which remain to be determined.

Not one of the insecticides used in our study caused DNA fragmentation in neuromuscular cells of L4 and pupae of the yellow fever mosquito, unlike what has been reported for different tissues of other insect species exposed to insecticides [41-43]. Although not detected in our study, cell death in the neuromuscular system may still occur in mosquito larvae and pupae, but a more detailed investigation is necessary to elucidate these changes.

## Conclusion

Deltamethrin and spinosad exhibited a high efficacy against *A. aegypti* L4 followed by imidacloprid. In contrast, azadirachtin exhibited a low potency and efficacy against larvae of the yellow fever mosquito and did not impair the swimming activity of L4 and pupae, unlike the neurotoxic insecticides. The overall reduction in the swimming activity may compromise the foraging and evasion of the exposed insects. The potential use of spinosad against mosquito larvae and pupae is reinforced by the results obtained for both the lethal and sublethal assessments.

## Competing interests

The authors declare that they have no competing interests.

## Authors’ contributions

Conceived and designed the experiments: HVVT, RACD, RNCG and GFM. Performed the experiments: HVVT, TVP and RACD. Analyzed the data: HVVT and RNCG. Contributed reagents/materials/analysis tools: HVVT, RNCG and GFM. Wrote and reviewed the paper: HVVT, RNCG and GFM. All authors read and approved the final version of the manuscript.
